# Commentary: Another way to skin a rare and dangerous cat

**DOI:** 10.1016/j.xjtc.2021.02.033

**Published:** 2021-02-27

**Authors:** Robert D.B. Jaquiss

**Affiliations:** Division of Pediatric Cardiothoracic Surgery, Department of Thoracic and Cardiovascular Surgery, University of Texas Southwestern Medical Center and Children's Medical Center, Dallas, Tex

**Central Message**

In pulmonary atresia/intact septum with bilateral coronary ostial atresia, antegrade coronary blood flow may be optimized by an aorto-right ventricular shunt, to the free wall or tricuspid annulus.

**Figure:**
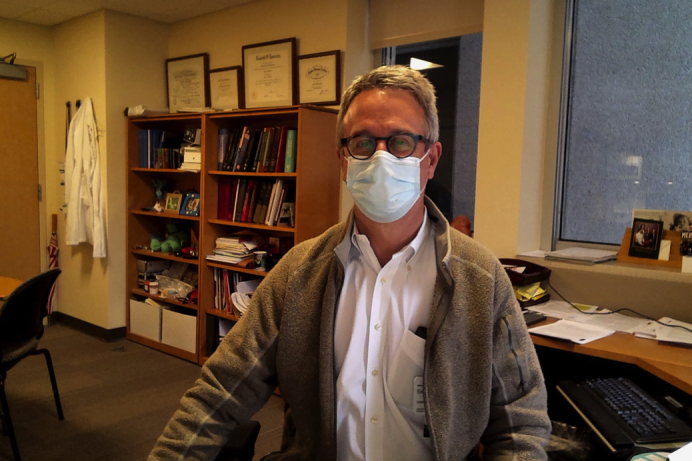
Robert D. B. Jaquiss, MD

See Article page 216.

In patients born with pulmonary valve atresia with intact ventricular septum (PA/IVS), right heart structures may be so hypoplastic that biventricular circulation cannot be achieved.^1^ In the most unfavorable manifestation of PA/IVS, the epicardial coronary arteries are stenotic (or proximally atretic) such that a significant amount coronary arterial flow is provided by the hypoplastic and hypertensive right ventricle via sinusoidal connections to the coronary arteries. This condition is termed “right ventricular dependent coronary circulation” (RVDCC), the most extreme form of which is bilateral coronary ostial atresia.^2^ In many centers, patients with PA/IVS with RVDCC are directed toward cardiac transplantation rather than single-ventricle palliation culminating in the Fontan circulation.^3^ Patients with RVDCC are at high risk of sudden death, particularly in the first few months of life, when pulmonary blood flow is provided by either a patent ductus arteriosus or a systemic to pulmonary shunt (SPS) with consequent diastolic hypotension. Often such patients demonstrate electrocardiographic evidence of myocardial ischemia, sometimes accompanied by elevations in serum levels of troponin or other markers of cardiomyocyte injury.

One approach to address the coronary insufficiency in RVDCC is to provide “antegrade” blood flow to the right ventricular cavity by creation of an aorto-right ventricle shunt (ARVS). This was initially described in an older child at the time of Fontan completion.^4^ Later, a small series was reported with placement of ARVS in 5 children at varying ages, the youngest of whom was 6 weeks of age and manifested coronary ischemia a few weeks after an SPS.^5^ More recently, Sakurai and colleagues^6^ described simultaneous placement of SPS and ARVS in a 6-week-old patient with bilateral coronary ostial atresia and ongoing myocardial ischemia.

In the present report, Said and colleagues^7^ described, in a similar patient, modifications of this approach, which provided bridge to transplant, albeit not without interval complications (2 separate courses of extracorporeal membrane oxygenation). Their modifications included anastomosis of the ARVS to the tricuspid annulus (after valvectomy) via the right atrium, and employment of allograft saphenous vein as conduits for both the ARVS and the SPS. The authors suggest that avoidance of anastomosis to the thickened right ventricular wall is preferrable to avoid anastomotic obstruction. However, such obstruction has not been reported in ARVS to date, and the widespread use of similar anastomoses in the modified Norwood operation suggests that most neonatal surgeons have overcome this issue. While anastomosis to the tricuspid annulus eliminates concern about tricuspid regurgitation after ARVS, closing the orifice with a patch as described by Sakurai and colleagues would seem to accomplish the same goal in a simpler fashion. Finally, the suggested superiority of allograft conduit in comparison with expanded polytetrafluoroethylene conduit remains speculative, in either SPS or ARVS application, and at present remains a “surgeon's choice” matter. Despite these minor quibbles, I applaud the authors for an inventive and skillful solution to an admittedly rare but potentially lethal variant of congenital heart disease.
